# Growth of Homogeneous Luminescent Silicon–Terbium Nanowires by One-Step Electrodeposition in Ionic Liquids

**DOI:** 10.3390/nano10122390

**Published:** 2020-11-30

**Authors:** Shibin Thomas, Jeremy Mallet, Bijal K. Bahuleyan, Michael Molinari

**Affiliations:** 1 Laboratoire de Recherche en Nanosciences, LRN EA4682, Université de Reims Champagne-Ardenne, Campus Moulin de la Housse, 51687 Reims, France; shibin.thomas@soton.ac.uk; 2 Department of General Studies, Yanbu Industrial College, Yanbu Al Sinaiyah 41912, Saudi Arabia; bijal@rcyci.edu.sa; 3 Institute of Chemistry and Biology of Membranes and Nanoobjects, CBMN UMR CNRS 5248, Université de Bordeaux, IPB Bordeaux, Allee Geoffroy Saint Hilaire, 33600 Pessac, France

**Keywords:** electrodeposition, silicon/terbium, nanowires, luminescence

## Abstract

An electrodeposition method for the growth of homogeneous silicon–terbium nanowires (NWs) with green light emission is described. The method involves template-assisted electrochemical co-deposition of Si/Tb NWs with 90-nm diameter from an electrolyte bath containing Si and Tb precursors in an ionic liquid (IL). This method of deposition is advantageous over other conventional techniques as it is relatively simple and cost-effective and avoids harsh deposition conditions. The deposited NWs are of uniform dimensions with homogeneous composition incorporating 10% of Tb and exhibit intense room temperature (RT) luminescence in the visible range due to Tb emission. These results were confirmed by combining classical characterization such as scanning electron microscopy (SEM) and photoluminescence (PL) performed on an assembly of NWs with spatially resolved experiments such as transmission electron microscopy (TEM) and cathodoluminescence (CL). This electrodeposition method provides an alternative and extremely simple approach for depositing silicon-rare earth nanostructures for optical and sensing applications.

Silicon-based optical materials in the form of one-dimensional (1-D) nanomaterials such as nanowires and nanotubes have gained huge scientific and technological interest due to their unique opto-electronic properties [1] with applications in various fields including photovoltaics [2], light emitting diodes (LEDs) [3], lasers [4], and sensors [5,6]. Nevertheless, as bulk crystalline silicon is an indirect band gap material, its optical properties remain limited and one solution to improve them is to couple silicon with rare earth (RE) ions or oxides of RE [7] which bring interesting emission properties to the system. Different types of samples are prepared either by direct incorporation of RE ions into the structures during the growth process such as physical or chemical techniques or by grafting RE complexes at the surface of the structure after preparation of the nanostructures [8,9]. Although Si–RE samples including nanowires (NWs) have been successfully prepared by different groups [10,11,12,13,14], the preparation of such systems remains constraining as it needs either harsh deposition conditions or different time-consuming steps limiting its scale up. Among the different RE ions, the trivalent terbium ion (Tb^3+^) is an important dopant for Si as it exhibits a large Stokes shift (separation between the excitation and emission maxima) [15,16], sharp luminescence emission in the visible wavelengths compatible with the fabrication of Si-based light emitters or Si-based solar cells [17], and a long lifetime in the order of the millisecond allowing optical sensing applications. So far, many studies incorporating Tb in silicon-related thin films have been performed [18,19,20,21], but the incorporation of Tb in Si NWs have not been well attended and the reports are very limited [14,22]. Whatever the techniques used to prepare Si–Tb samples, the processes remain complicated and constraining as in the case of porous silicon (p-Si) doped with Tb^3+^ ions [22,23] or while using a vapor–liquid–solid (VLS) mechanism to fabricate Tb-doped silica nanowires [14]. Indeed, in the case of p-Si, the growth method is a two-step process, and at the end, because of the lack of stability of p-Si and its fragile nature, their use in opto-electronic devices is limited and, for the VLS technique, the need of a metal catalyst (Au or Fe) to initiate the reaction could alter its electronic properties [24]. Also, the precise control of the NW diameters and of the Tb ion distribution [25], which ensures homogeneity of the optical properties, is difficult for these different techniques. To overcome these limitations, electrodeposition has recently emerged as a simple and low-cost alternative for the growth of semiconducting nanostructures. It facilitates the low temperature (<100 °C) deposition of NWs, and it is easily transposable to an industrial scale. In particular, the use of ionic liquid (IL) as electrolyte solvents and the use of templates to precisely control the dimensions of Si-related NWs have been explored. Electrodeposition of amorphous Si NWs and nanotubes from ILs has been previously reported by our group [26,27,28] and others [29,30]. However, the possibility of co-depositing Tb with Si to achieve Si/Tb NWs have not been investigated although we developed a method to incorporate RE within Si thin films [31]. Herein, we demonstrate a one-step template-assisted electrodeposition technique using ILs for the growth of Si/Tb NWs with a diameter of 90 nm. Using nanoscale structural and optical characterization techniques, we show that our method allows for obtaining NWs with homogeneous diameters and composition along the NWs with 10% Tb content. The NWs exhibit visible emission bands of the Tb at room temperature (RT) with a millisecond scale lifetime and a homogenous luminescence along the NWs.

The Si/Tb NWs were prepared using an electrolytic bath containing 1-butyl-1-methylpyrrolidinium bis(trifluoromethanesulfonyl)imide (Py_1,4_[TFSI]), SiCl_4_ (0.01 M), and TbCl_3_ (0.01 M). Nanoporous polycarbonate (PC) membranes with 90-nm pore diameter served as the template for the deposition. Prior to use, a thin layer of gold (ca. 200 nm) was sputtered on one side of the PC membrane to make the electrical contact. More details of the electrochemical experimental setup are explained in our previous report [32]. All the electrochemical potentials mentioned here are referenced to a Pt quasi-reference electrode. The structural, morphological, and compositional characterization of the Si/Tb NWs were obtained by scanning electron microscopy (SEM) (JSM-7900f, JEOL Ltd., Tokyo, Japan) and transmission electron microscopy (TEM) (2100F, JEOL Ltd., Tokyo, Japan) equipped with energy dispersive X-ray (EDX) spectrometer and a Gatan cathodoluminescence (CL) setup (Gatan Inc., Pleasanton, CA, USA). The photoluminescence (PL) spectrum and lifetime were obtained on a FLS1000 system (Edinburgh Inc., Edinburgh, UK) with an excitation at 325 nm and a nitrogen cooled silicon photomultiplier tube (PMT) for detection. The quantum yield measurements were performed with a Horiba-Jobin Yvon Fluorolog 3 System with a xenon lamp as a source. For the quantum yield, classical fluorescence measurement protocols [33] were performed following the procedure setup by the European and American standardization laboratories using fluorescein as a standard sample.

A schematic representation of the electrochemical setup used for electrodeposition is shown in Figure 1. Prior to performing electrodeposition of the NWs, an investigation on the electrochemical behavior of the precursors in IL was carried out by cyclic voltammetry (CV) studies. The CV was used to gain important information on the electrochemical processes at the interface and to determine the deposition potential to be used for the growth of Si/Tb NWs. First, the electrochemical behavior of individual precursors, i.e., SiCl_4_ and TbCl_3_, in Py_1,4_[TFSI] was studied by performing their CVs to evidence the possible deposition of the metallic species, and then, an electrolyte solution containing a mixture of these two precursors was analyzed to study the electrochemical feasibility of co-depositing Si and Tb simultaneously. The red and blue curves in Figure 2 shows the individual CVs of 0.01 M SiCl_4_ and 0.01 M TbCl_3_, respectively, in Py_1,4_[TFSI] at 50 °C. Details on the electrochemistry of individual precursors in Py_1,4_[TFSI] are described in our recent reports [31,32], which proves the possibility of depositing Si and Tb in their elemental form. An electrolyte solution containing a mixture of these two precursors was then analyzed by CV.

The black curve in Figure 2 shows the CV of an electrolyte solution containing 0.01 M SiCl_4_ and 0.01 M TbCl_3_ in Py_1,4_[TFSI] at 50 °C. The peak centered at −2.1 V on the red curve corresponds to the Si ions reduction into Si metal, whereas the deposition peak corresponding to Tb^3+^ reduction on the blue curve starts at −2.5 V but is partially hindered by the Py_1,4_[TFSI] degradation. In the presence of both Tb^3+^ and Si^4+^ ions (black curve), the cathodic peak corresponding to Si^4+^ reduction is observed as an intense reduction wave at −2.6 V, meaning that the coexistence of these two ions in ILs causes changes in their individual electrochemical behavior. The third reduction plateau observed at −3.0 V corresponds well to the reduction potential of the Tb^3+^ ions. It might also contain contribution from the reduction of Si^4+^ ions, as the second peak corresponding to Si^4+^ reduction is also observed at this potential range. In addition, a stripping peak around −0.4 V is observed in the anodic scan, the origin of which is unclear and beyond the scope of this study. It is evident from the CV studies that Si and Tb could be co-deposited as NWs by applying an appropriate potential in Py_1,4_[TFSI] electrolyte with a controlled precursor concentration. After the CV studies, potentiostatic electrodeposition was performed in the electrolyte containing 0.01 M SiCl_4_ and 0.01 M TbCl_3_ in Py_1,4_[TFSI] by applying −3.2 V at 50 °C. The chosen potential is well above the cathodic reduction potentials of both Si^4+^ and Tb^3+^ ions and hence provides sufficient overpotential for both ions to be reduced into the nanopores. Regarding the synthesis yield of the process, it could be noted that, with the used parameters (pore diameters and density), almost all the pores are filled with NWs, leading to a yield close to 100% compared to the number of pores. Nevertheless, dissolution of the PC membrane, necessary to study the NW properties, led to a loss of NWs within the solvent (or breakage during centrifugation). After dissolution of the membranes and cleaning of the NWs, they were collected either on a TEM grid or directly on a Si (100) substrate for further characterization. SEM analysis (Figure 3a) shows a typical network of Si/Tb NWs, slightly flexible and interconnected. The flexibility of the NWs could be due to their amorphous nature as previously observed for silicon-based NWs or films prepared by electrodeposition [27,32] and confirmed by the electronic diffraction pattern obtained by TEM on a single NW (Figure 3c inset). From the microscopic analysis, the diameters of the NWs are uniform and around 90 nm, which corresponds well to the diameter of the nanopores of the template. To study the chemical composition and to confirm the successful incorporation of Tb in Si NWs, EDX analysis has been performed on a bunch of NWs in SEM and on isolated NWs in TEM. The EDX spectrum (Figure 3b) shows signals corresponding to Si, Tb, and O. The presence of both Si and Tb confirms co-deposition of them during the electrodeposition and proves their coexistence in the NWs. As already observed for other Si-based structures prepared with ILs, the oxygen signal arises from the surface oxides formed on the NWs when they are exposed to air, owing to the high affinity of Si and Tb towards oxygen, forming their sub-oxides (SiO_2_ for Si, and Tb_2_O_3_ or Tb_4_O_7_ [34] for Tb). To gain further insights into the structural and compositional quality of Si/Tb NWs, TEM measurements were then performed on a single NW. TEM image of a representative Si/Tb NW (Figure 3c, left) confirms uniform diameter of 90 nm throughout its length and shows the presence of fine nanoscale grains indicative of a perfect bottom-up electro-reduction process. To confirm the homogeneity in the distribution of different elements along the NW, EDX analysis was carried out in elemental mapping mode (Figure 3c, right). It reveals a quite homogeneous distribution of Si and Tb along the whole length of the NW. It indicates that the electrochemical co-deposition results in uniform deposition of both Si and Tb and maintains the elemental ratio between them throughout the deposition time. This is of particular importance when it comes to incorporating RE into Si as other growth mechanisms could lead to aggregation of Tb within Si, which is detrimental for optical applications due to a reduced number of optically active centers [25]. In addition to Si and Tb, oxygen is also uniformly present throughout the length of NW, confirming the oxidation of Si and Tb. From the different EDX spectra acquired in TEM and SEM, the peak ratios were used to estimate the atomic percentage of Tb and O in the NWs, which were found to be 10 ± 2% and 12 ± 5%, respectively, whatever the spot position on the NW. Such a high concentration of Tb with homogeneous distribution over the NWs, without clustering, is advantageous in obtaining efficient green luminescence as the cross-relaxation processes occurring at high dopant concentrations favor green emission over blue emission [35]. For oxygen, as we are working in a glove box and as already observed for the pure Si NWs prepared with the same techniques [32], oxidation appeared after membrane dissolution and after exposure to air. Then, oxidation should mainly occur on the surface, even if some oxygen atoms should diffuse within the NW volume.

These structural and chemical results confirm that the electrochemical co-deposition is a quite efficient way of producing Si/Tb NWs. A major advantage of this technique is that the Tb incorporation in Si NWs is achieved in a single step, as the growth of Si and Tb occurs together during electrodeposition. Additionally, this bottom-up fabrication method results in uniform incorporation of Tb both in the core and on the surface of Si NWs. Furthermore, the Tb is incorporated into Si, which is amorphous in nature and is important for the optical properties of Si/Tb. Indeed, it has been reported that a-Si offers higher solubility to RE ions compared to c-Si and that the thermal quenching of RE luminescence is found to be lower when the host matrix is a-Si [7]. Following the structural characterization of the NWs, optical experiments have been performed on Si/Tb NWs. First, PL emission properties were studied using an excitation line at 325 nm as it matches well with the absorption of Tb [36]. A typical PL spectrum (Figure 4a) shows four main luminescence bands between 450 and 650 nm, which originates from the radiative transitions between the intra 4f energy levels of Tb^3+^ ions in Si NWs. The observed transitions are ^5^D_4_-^7^F_6_ (489 nm), ^5^D_4_-^7^F_5_ (544 nm), ^5^D_4_-^7^F_4_ (585 nm), and ^5^D_4_-^7^F_3_ (620 nm). The most intense transition is ^5^D_4_-^7^F_5_, resulting in a strong green luminescence. The relatively wide line width of the emission bands is because the Tb^3+^ ions are incorporated in an amorphous Si matrix. The presence of the Tb^3+^ luminescence shows that an energy transfer between the Si matrix and Tb^3+^ ions occurs. This energy transfer could be from the Si matrix which has been shown to emit in the visible range [32] or from oxygen-mediated excitation of the lanthanides [37], where Tb^3+^ ions can be easily coordinated by oxygen atoms present in the NWs, as shown previously. Due to the cross-relaxation processes occurring at high concentrations [38], the Si/Tb NWs with a Tb content of 10 at.% shows only emissions corresponding to ^5^D_4_ to ^7^F_j_ transitions, which is indeed characterized by a strong green emission. Time-resolved PL experiments were also carried out to gain information about the carrier lifetime. The resulting decay curve (Figure 4a inset) is a single exponential with a calculated lifetime of 2.3 ms which is in the range of Tb^3+^ emitting structures [38]. This high value indicates that the quenching mechanisms and the nonradiative paths that could have occurred during the synthesis are not relevant here. It should be noted that this long lifetime obtained is from Tb^3+^ emission and is not related to the pure Si nanowires as the lifetime of Si nanowires is shorter [32]. Such a high lifetime is of particular importance for applications such as sensors as it would allow a proper response time. For instance, for bio-labelling applications, such lifetime values are much longer than the lifetime of the biological samples, which allows to discriminate different signals. Fluorescence measurements were performed on the NWs to estimate the quantum yield (QY) values. The QY of Si/Tb NWs is found to be 2 ± 1%, which is low but a reasonable value for Si-related materials. However, it should be noted that the QY results were obtained from the as-deposited NWs and it could be improved by annealing the NWs to increase the atomic diffusion and to optically activate the Tb^3+^ ions. As the PL signal originates from an assembly of NWs and do not give information about the homogeneity of the emission, CL experiments on isolated NWs were performed. Apart from the better spatial resolution of a few nm [39], CL is also interesting as it gives information about the optical emission from materials under electronic excitation. Figure 4b shows the correlated CL/TEM image of two Si/Tb NWs stacked together. The typical CL spectrum (Figure 4b inset) obtained from the NWs is similar to the PL spectrum showing different Tb^3+^ lines. Despite an electronic excitation of 100 kV, only a small additional blue broad band appears, which should originate from SiO*_x_* defects appearing under irradiation. The CL spectrum with the important Tb^3+^ bands confirms the robustness of the electrodeposited Si/Tb NWs. To confirm the NW homogeneity, a panchromatic CL image has been recorded between 400 and 800 nm corresponding to the visible emission of Tb^3+^ ions. Figure 4b shows that the Si/Tb NWs are homogeneously emitted along their whole length, which also shows the high homogeneity of Tb distribution. This point is of prime importance for applications in opto-electronics or sensing. It is important to note here that the strong luminescence emissions have been obtained at RT from the as-deposited Si/Tb NWs without any other treatments. In most of the conventional Si/Tb materials, a high-temperature annealing treatment is unavoidable to optically activate the Tb^3+^ ions and to obtain efficient luminescence [35,38]. However, it is shown here that the simple oxidation of Tb in air is sufficient to get strong visible luminescence from as-deposited NWs. When the Tb-incorporated Si is prepared in the form of NWs, there is obviously an enhancement of surface area which is exposed to the air leading to a high degree of oxidation and consequently resulting in an increased number of emitting centers. The electrochemical co-deposition technique is therefore highly advantageous as it can produce a dense array of Si/Tb NWs with homogeneous distribution of the Tb in the NWs. Even though there exists only a few papers regarding the incorporation of Tb in Si or in SiO_2_ NWs [14,22], it seems that our electrodeposition method allows to produce a quite robust network of NWs with significantly high concentration of Tb but still exhibiting room temperature emission. On smaller SiO_2_ NWs grown by chemical vapor deposition (CVD) (diameters between 15 and 30 nm), the paper from Lin et al. reported a 4% Tb incorporation with room temperature PL showing the interest of CVD-related methods. Nevertheless, they are growing SiO_2_ NWs instead of Si NWs, and this could limit applications in many fields as the SiO_2_ NWs are insulating.

In conclusion, a one-step electrodeposition method for the synthesis of Si/Tb NWs using ILs has been demonstrated. This template-assisted electrodeposition allows for obtaining Si/Tb NWs with good structural and compositional quality, characterized by homogeneous distribution of Tb in the NWs. The NWs are found to be amorphous and possess uniform diameter and length. The Tb content in the NWs is estimated to be 10 ± 2 at.%. The PL and CL experiments confirmed strong light emission at visible wavelengths from the as-deposited Si/Tb NWs and have a lifetime in the ms range. The strong luminescence at RT and the associated lifetime in the ms range confirm the quality of the electrodeposited NWs for application in modern opto-electronics. The electrochemical co-deposition technique elaborated here demonstrates a simple and low-cost alternative for the synthesis of RE-incorporated Si nanostructures.

## Figures and Tables

**Figure 1:**
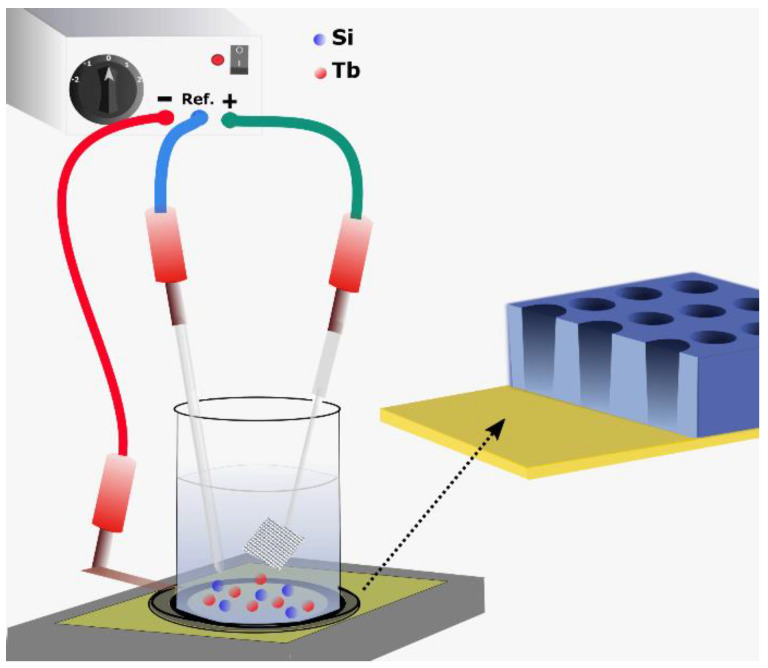
A schematic representation of the electrochemical setup used for electrodeposition of Si/Tb nanowires (NWs).

**Figure 2:**
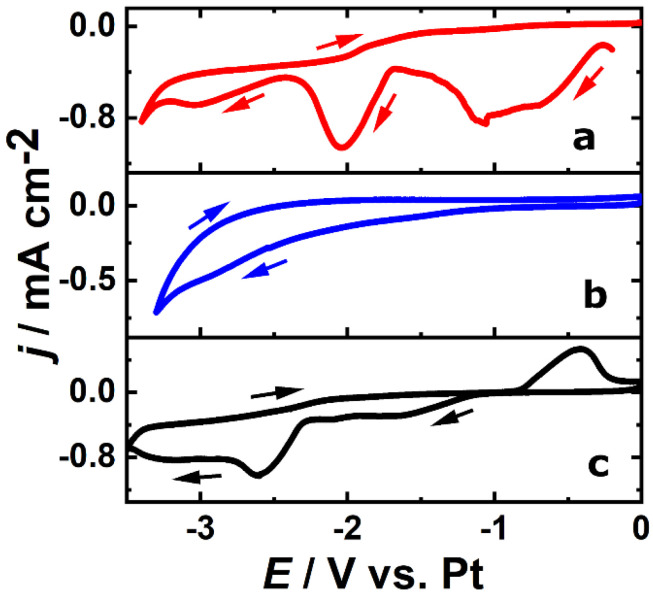
The cyclic voltammetry (CV) of 0.01 M SiCl_4_ (**a**), 0.01 M TbCl_3_ (**b**), and 0.01 M SiCl_4_ and 0.01 M TbCl_3_ (**c**) in Py_1,4_[TFSI] at 50 °C: The scan rate is 10 mV s^−1^.

**Figure 3:**
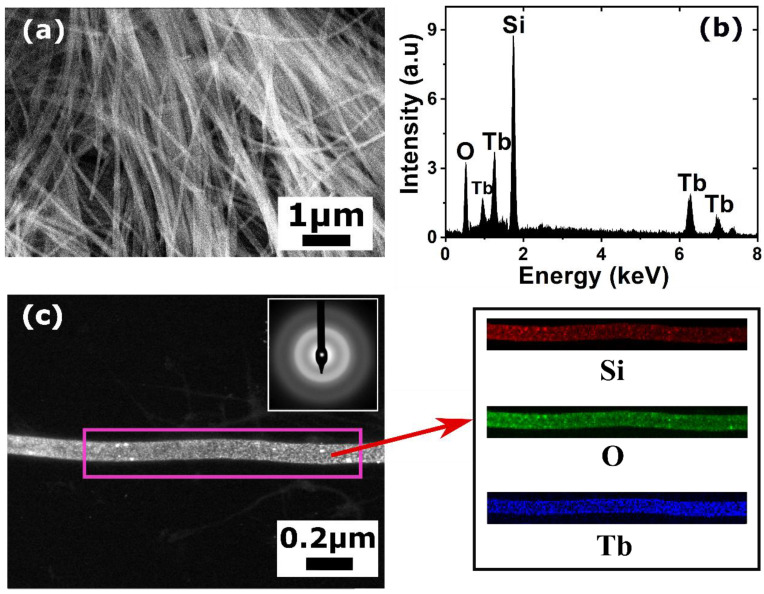
(**a**) Scanning electron microscopy (SEM) image of the Si/Tb NWs; (**b**) Energy dispersive X-ray (EDX) spectrum obtained from the Si/Tb NWs; (**c**) Transmission electron microscopy (TEM) image of a single Si/Tb NW and the EDX elemental mapping of Si, O, and Tb on the NW: the inset shows the electron diffraction pattern.

**Figure 4:**
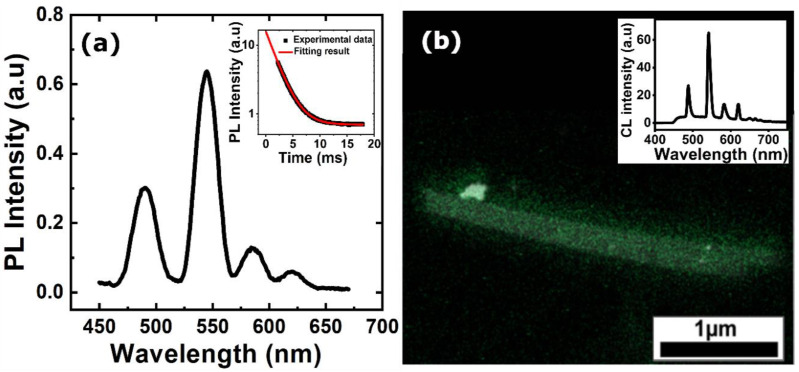
(**a**) Photoluminescence (PL) emission spectrum obtained at room temperature (RT) from the Si/Tb NWs. The inset shows the PL decay curve; (**b**) The correlated TEM/cathodoluminescence (CL) image of two Si/Tb NWs stacked together. The inset shows the CL spectrum recorded from Si/Tb NWs.
